# Fibromyxoid Nephrogenic Adenoma in the Ureter

**DOI:** 10.1089/cren.2018.0037

**Published:** 2018-07-01

**Authors:** Benjamin M. Dropkin, Giovanna A. Giannico, Peter A. Reisz, David F. Penson, Ryan S. Hsi

**Affiliations:** ^1^ Department of Urologic Surgery, Vanderbilt University Medical Center, Nashville, Tennessee.; ^2^ Department of Pathology, Microbiology, and Immunology, Vanderbilt University Medical Center, Nashville, Tennessee.

**Keywords:** fibromyxoid, nephrogenic adenoma, ureter, ureteral neoplasm

## Abstract

***Background:*** Nephrogenic adenoma is a benign lesion found in the genitourinary tract, often at sites of prior inflammation, and is characterized by tubular, papillary, or tubulopapillary structures. It is thought to arise from distal migration and implantation of renal tubular cells into the renal pelvis, ureter, bladder, or urethra. These tumors often resemble malignant neoplasms. Morphologic variants include small tubules, signet ring-like pattern, papillary formations, flat pattern, and vessel-like structures. A fibromyxoid variant was first described in 2007. Here, we present the first known cases of fibromyxoid nephrogenic adenoma of the ureter.

***Case Presentations:*** A 79-year-old white man presented with asymptomatic right hydroureteronephrosis to the level of the mid-ureter with associated right ureteral wall thickening found on surveillance CT scan for lymphoma. A 59-year-old white man presented with a right ureteral stricture after ureteroscopic ureteral injury and underwent effective robotic ureteroureterostomy. Pathology analysis in both cases revealed fibromyxoid nephrogenic adenoma.

***Conclusion:*** Fibromyxoid nephrogenic adenoma may occur in the ureter. Knowledge of this rare tumor is important for urologists and pathologists to prevent misdiagnosis and overtreatment of a typically benign process.

## Introduction and Background

Nephrogenic adenoma is a benign lesion found in the genitourinary tract, characterized by tubular, papillary, or tubulopapillary structures, and thought to arise from migration of renal tubular cells with distal replantation.^1^ These tumors often resemble malignant neoplasms and can arise in the renal pelvis, ureter, bladder, or urethra. Morphologic variants include small tubules, signet ring-like pattern, papillary formations, flat pattern, and vessel-like structures.^2^ A new variant consisting of focal elements of nephrogenic adenoma in a background of compressed spindle cells in a fibromyxoid matrix was first described in 2007.^2^ This new fibromyxoid variant was identified in specimens from transurethral resection of the prostate, bladder biopsies, urethral biopsies, cystoprostatectomy, and radical nephroureterecomy.^2^ We present two novel cases of fibromyxoid variant nephrogenic adenoma arising from the ureter.

## Presentation of Cases

### Case 1

**FIG. 1.**
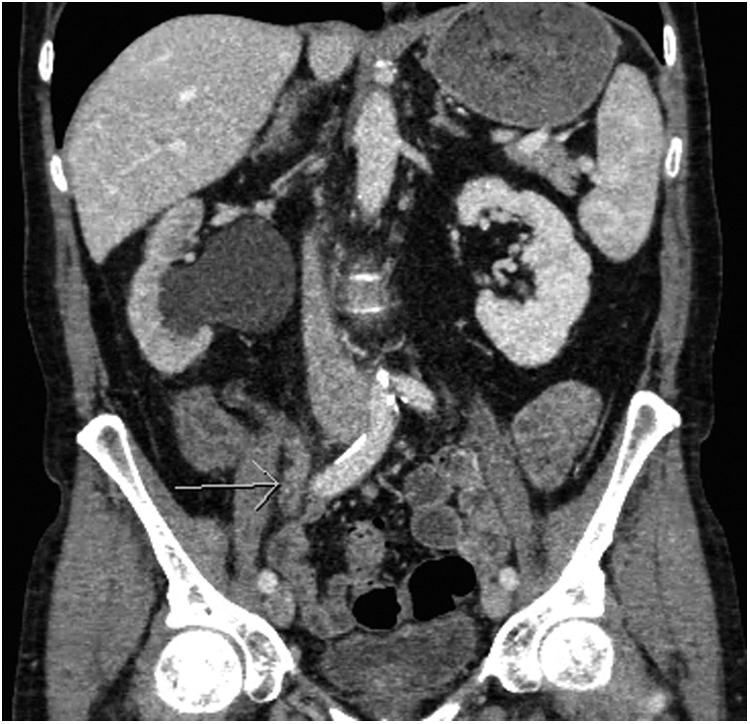
Right hydronephrosis secondary to a right ureteral mass.

**FIG. 2.**
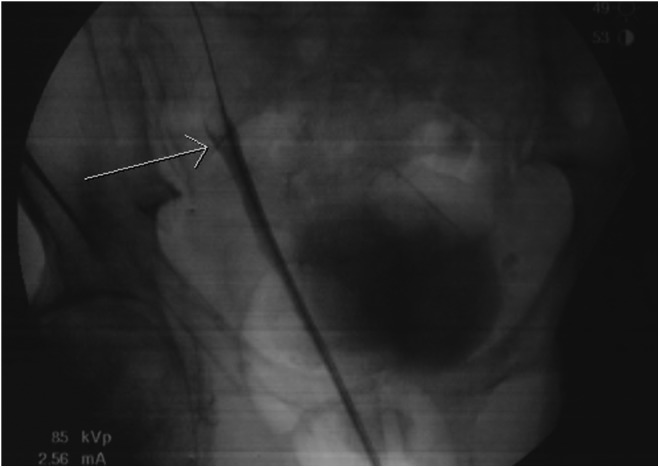
Retrograde pyelography showing right ureteral mass with goblet sign (*arrow*).

**FIG. 3.**
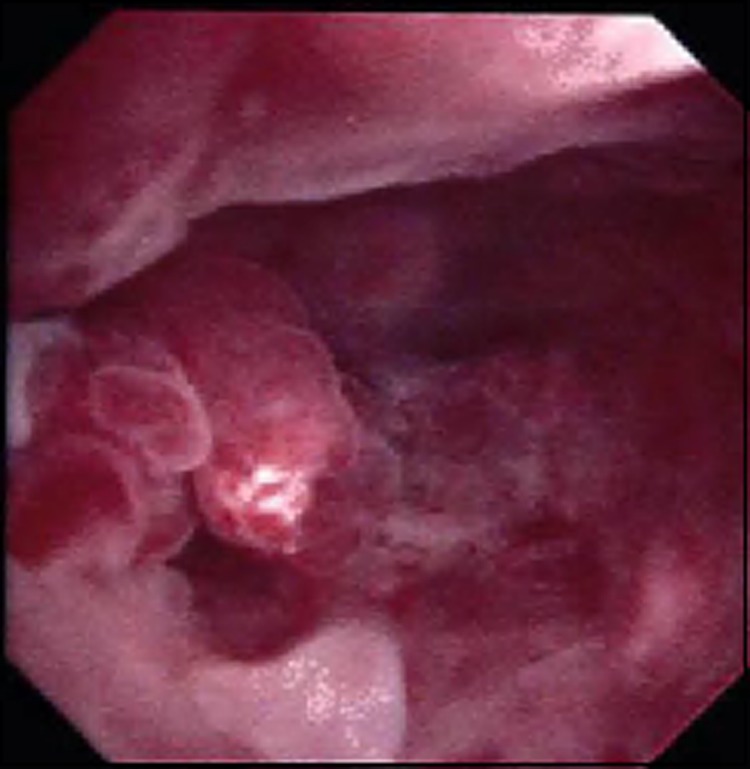
Intraoperative picture of right ureteral mass during retrograde ureteroscopy.

**FIG. 4.**
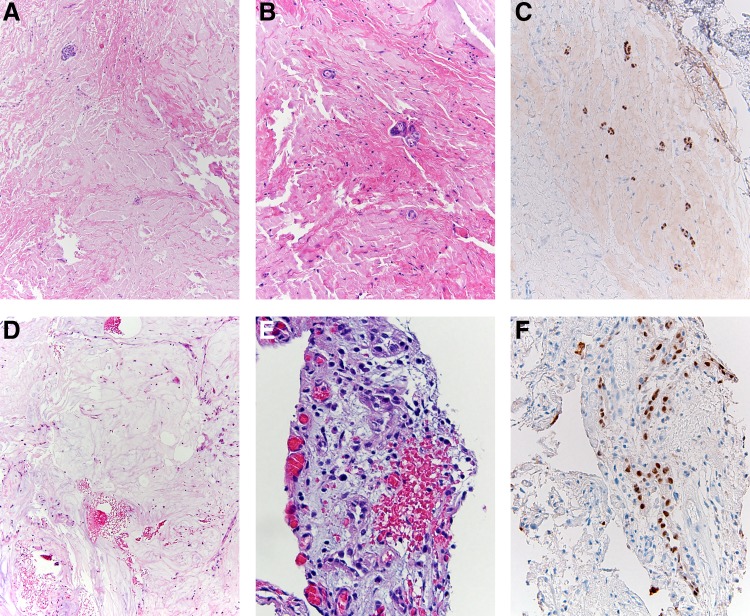
Histologic images of fibromyxoid nephrogenic adenoma. Both cases (case 1, **A–C** and case 2, **D–F**) showed rare minute tubular structures within an abundant fibromyxoid stroma. Note the bland cytologic features **(E)** with low nuclear–cytoplasmic ratio and lack of hyperchromasia and pleomorphism. Nuclear expression of PAX-8 was present by immunohistochemistry **(C, E)**. Magnification, **A–D**, × 100; **E, F**, ×200.

A 79-year-old man with a distant history of colon cancer treated with surgery and radiation and diffuse large B cell lymphoma presented with asymptomatic right hydroureteronephrosis to the level of the mid-ureter with associated right ureteral wall thickening found on surveillance CT scan for lymphoma (Fig. 1). With cystoscopy, the right ureteral orifice could not be identified because of prior pelvic radiation. Antegrade ureteroscopy facilitated biopsies taken with Piranha forceps (Boston Scientific, Marlborough, MA). Pathology analysis showed small fragments of denuded urothelial mucosa with small submucosal glandular structures composed of cuboidal cells with low nuclear to cytoplasmic ratios, lightly eosinophilic cytoplasm, mild nuclear pleomorphism, and small nucleoli. No mitoses were identified. The glandular structures stained positively for cytokeratin AE1/AE3 and PAX-8 and negative for GATA-3, suggestive of nephrogenic adenoma. Repeat biopsies through retrograde ureteroscopy (Figs. 2 and 3) showed similar changes with rare small tubular structures within a fibromyxoid stromal background. Repeat immunohistochemical studies showed the small glands to stain positively with PAX8, highlighting rare foci of nephrogenic adenoma of the fibromyxoid type (Fig. 4). The patient elected for long-term management with interval ureteroscopic tumor debulking and ureteral stents. Retrograde ureteroscopy facilitated effective tumor debulking, which was achieved with five grasps of a 1.9 French Zero Tip Nitinol basket (Boston Scientific).

### Case 2

A 59-year-old male with no nephrolithiasis history underwent right ureteroscopy and laser lithotripsy for a right mid-ureteral stone. The procedure was complicated by a urinoma managed with an indwelling ureteral stent and retroperitoneal drain placement. He ultimately developed a mid-ureteral stricture. MAG-3 lasix renogram showed 50% split function. He then underwent effective robotic right ureteroureterostomy. Pathology analysis revealed scattered minute tubular structures within a fibromyxoid stroma and immunohistochemistry was positive for PAX8, consistent with fibromyxoid nephrogenic adenoma.

## Discussion and Literature Review

Nephrogenic adenoma is thought to arise from migration of renal tubular cells with distal replantation.^1^ Nephrogenic adenomas typically feature a cuboidal to flat epithelium in a single or multiple layers with frequent hobnailing. By immunohistochemistry, nephrogenic adenomas express cytokeratin 7, CD10, alpha-methylacyl-coenzyme A racemase, PAX2, and PAX8. In a series of 80 cases, these tumors arose in the bladder (55%), urethra (41%), and ureter (4%).^3^ Clinically, nephrogenic adenoma of the ureter may be associated with prior inflammation from urothelial stone trauma and can present with symptomatic or asymptomatic obstruction. Often it can resemble carcinoma radiographically. Treatment is aimed at preserving renal function and may include resection, chronic stent placement, and nephrostomy tube placement. Follow-up studies have indicated that nephrogenic adenomas in the genitourinary tract can recur after resection but do not progress to carcinoma or metastasize.^4^ Two case reports have described bladder tumors that appear to have undergone malignant transformation from nephrogenic adenoma to adenocarcinoma, underscoring the importance of continued follow-up for patients with nephrogenic adenoma.^4^

## Conclusion

We have presented two novel cases of fibromyxoid variant nephrogenic adenoma arising from the ureter. Knowledge of this rare tumor is important for urologists and pathologists to prevent misdiagnosis and overtreatment of a typically benign process.

## Abbreviation Used

CT: computed tomography
